# On the Diseases of India

**Published:** 1830-07-01

**Authors:** 


### XXIV.

ON THE DISEASES OF INDIA. By JAMES ANNESLEY, Esq. Vol. II. Continued from Vol. XI. of this Journal, page 512.

I. *Cursory Remarks on the Presence of Worms in the Large Bowels.*

Intestinal worms are very frequently met with between the tropics. Among Europeans they are generally the consequence of torpid bowels, or accumulations of morbid secretions in the alimentary canal. Ascarides, Lumbrici, and Tæniæ are the most frequently observed. Among the natives there are scarcely any free from these parasitical animals.

"It will generally be found amongst the natives of warm climates, and among those Europeans who have been much weakened by their residence in them, that the secretions, which form the principal part of the fæcal discharge, are seldom thrown off from the mucous surface of the large bowels in so quick a manner as in the robust individual who enjoys an energetic state of the circulation and of all the organic functions; and being thus retained, they form at least the soil in which worms are reared, whatever may be the primary source from which these creatures proceed. Hence the importance of endeavouring to prevent the retention of morbid secretions and fæcal matters, and to impart energy to the digestive functions generally.

"The possibility of worms perforating the parietes of the intestines has been argued for by some pathologists, and denied by others. Without pretending to decide the question, we shall adduce the particulars of a case which came before us, in which the parietes of the bowel must have been perforated by them; but whether the perforation was effected previous to inflammation and ulceration having been excited in the part of the intestine in which they were lodged, or subsequently to that event, is a point which cannot be determined, from the imperfect history of the case, to which we were called only in its last stages.

"CASE—*In which Lumbrici passed from an opening at the Umbilicus.*

"*August* 5*th*, 1820.—J. W———, four years of age, according to the account furnished by his father, began to complain, about three months since, of swelling and hardness about the umbilicus, with pain on pressure. Opening medicines were prescribed by the medical man in attendance; and afterwards, finding that the tumour did not subside from their operation, poultices were applied. From the middle of April, the time at which the hardness was first detected, until the end of July, it gradually increased. The bowels, however, were always regular, even without the assistance of medicine, and the appetite was unimpaired. During July the swelling had increased considerably, was fluctuating, and slightly inflamed. The child's temper became irritable, and considerable symptomatic fever, with loss of appetite and cerebral irritation, supervened. Animal food was now abstained from, and saline diaphoretics and laxatives were given. On the 1st of August the abscess broke through two openings in the umbilicus, and discharged a great quantity of thick offensive matter. The usual dressings were applied. On the 2d, about a pint of yellowish watery fluid was discharged, with some thick offensive matter, similar to that which passed on the preceding day; and as a substance appeared to protrude through the aperture, which the father of the child fancied was the bowel itself, he became alarmed, and sent for us. We immediately drew from the opening two large lumbrici. This was the first time of our seeing the case. After this the child lived several days: the fæces, with eight or nine large lumbrici, passed through the opening at the umbilicus, and very little by the anus, during this period.

"*On examination*, the lower part of the ilium was found obstructed, its convolutions agglutinated together, and its canal, in parts, constricted to the size of a goose-quill. It presented no marks of recent inflammation, and was of a pale colour, both externally and internally. The agglutinated mass of small intestines adhered also to the abdominal parietes, around the umbilicus; and one of the most superficial convolutions of the intestine had an ulcerated opening through it, communicating with the external aperture at the umbilicus. The other abdominal viscera were natural in appearance."

In respect to the treatment, Mr. Annesley has not any thing new to offer. After proper purgation has been pursued to clear away the nidus of the worms, he was in the habit of prescribing enemata of castor oil and spirit of turpentine with great advantage. After this tonics were ordered. In cases of tænia, the oil of turpentine was advantageously given by the mouth, as also the bark of the pomegranate-tree.

### II. Hemeralopia, or Night-blindness.

This curious affection is very frequent between the tropics—especially among the natives of India. Mr. Annesley conceives that whatever be the peculiar condition of the sensorium or retina, in hemeralopia, the cause is debility, "accompanied with accumulations of morbid secretions in the primæ viæ, more particularly in the cæcum and colon, together with torpid function of the liver and stomach." The disease among the natives is usually induced by insufficient nourishment and inattention to the bowels. He has frequently found a well-regulated diet and purgative medicines sufficient for the removal of hemeralopia without any other remedy. Among Europeans the purgative medicines are essentially necessary, and they generally bring away copious, offensive, dark-coloured gelatinous stools, when the bowels become more sensible to purgatives, and then smaller doses suffice. Among debilitated Europeans and natives, generous diet and tonics are afterwards necessary, in order to prevent the generation of worms.

### III. Further Remarks on Accumulations of Morbid Matters in the Bowels, as a Cause of Nervous and other Ailments.

Our author commences this sub-section by remarking that, when the cæcum and colon become loaded, they disturb other viscera mechanically by pressure. Thus the right iliac veins and nerves are pressed upon by the distended cæcum, hence, he thinks, pains of the right lower extremity, or even partial paralysis. When the collections take place in the sigmoid flexure of the colon, the same phenomena take place in the left side.

"In addition to these symptoms, patients thus circumstanced frequently complain of pains in the loins, with occasional disorder of the urinary secretion, which is generally of a deeper colour than natural, and either depositing a very thick sediment, or exhibiting a very thick, mucous-like cloud, or both. When the fæcal accumulations are carried to the greatest height, then, in addition to the above ailments, or even independently of them in some cases, an œdematous state of the lower extremities supervenes, with an inability to use them, or at least a difficulty in subjecting them to the least voluntary exertion."

Mr. A. does not deny that there may be other causes for these symptoms; but merely asserts that this is one cause. He thinks the same collections of morbid matters are often the cause of rheumatism and gout. The functions of the liver are deranged in this way—but hepatic disorder is not seldom the precursor and cause of the intestinal accumulations. The distention of the bowels by flatus or more ponderous materials, will also embarrass the function of the lungs and the action of the heart. Indeed our author is inclined to trace almost all the diseases of the liver to this loaded state of the bowels—having, no doubt, his worthy chylopoietic master, of Bartholomew's, in view. It need hardly be added that the *methodus medendi* is reiterated purgation of the most active kind. The following is one of the best and most interesting cases which Mr. A. has adduced under this head. We shall give it unabridged.

*Case of Accumulations in the Cæcum and Colon, occasioning Anomalous symptoms.*

"Mrs. ———, aged about 27, had long been subject to daily paroxysms of fever; her hands were dry and white, as if powder lodged in the pores of the skin; the nails so brittle as not only to break easily, but to chip off in small flakes in every part of them. She had constant heaviness and noise in the head, with extreme pain supervening occasionally at the back of the neck, also weakness of sight, and a morning sleep invariably threw her into profuse perspirations. For these complaints medical advice had frequently been resorted to, but without advantage. Towards autumn the disorder assumed an intermittent character, and in the second week of October, 1822, medical advice was again called in.

"Her side was now submitted to the pressure of the hand, but as it was examined only to the termination of the ribs, she felt but trifling inconvenience from the touch; between the false ribs, however, and the umbilicus, the pressure of the hand occasioned the most acute pain; and her medical attendants immediately pronounced her liver diseased. Morning perspirations continuing, she was ordered to rise as soon as she awoke from a first sleep, however early it might be. This was of little avail, and decoction of bark was administered, which checked the perspirations for a time, but affected the head, and occasioned so much pain in the throat and stomach, attended with a burning and smarting sensation, that she was obliged to discontinue it. These sensations she began to feel after taking the first draught; and the slighest exertion, even lifting a chair, or walking across the room, would produce such extreme dryness in the throat as frequently to compel her to take some fluid to enable her to speak.

"She soon afterwards experienced increasing pain in the side and head; her spirits fluctuated, and sometimes became so depressed as to terminate in hysteric affections.

"She was now quite unable to lie upon her right side, as the least pressure occasioned acute pricking pain between the short ribs and the hip; and when lying upon the left side she had the sensation of heaviness and a dragging from the right; also, according to her own words, a pain down the middle of the body, from the chest to the umbilicus, as if the position in which she was placed had occasioned the liver to press upon the alimentary canal; she, therefore, invariably slept upon her back for many weeks. She also felt great difficulty in using her right arm, as the use of it produced heat and pain in the side and chest.

"Still her appetite was tolerably good, but she frequently felt compelled to sleep after dinner, particularly if she dined late; and she always found herself more comfortable when she divided the day so as to have her meals (breakfast, dinner, and tea) at nearly equal distances of time.

"In the beginning of February 1823, she stooped one day hastily to tie her shoe: this exertion soon proved to her how much the malady had increased. Acute pains succeeded, and in the night a violent attack of spasm. Some time after this she stooped suddenly to the right side, when she instantly suffered 'most severe pain in the soft part of the body, between the ribs and hip, with the addition of feeling near the groin as if a horny substance were piercing the intestines; and spasms succeeded in the night, as before.' She now every day felt increasing indisposition, weakness in the knees and ancles, a sensation of fulness in the right leg, with general debility; and the thumb of the right hand suddenly swelled, and became entirely useless.

"The direction that the pains of which she complained now took, was from 'the throat to the abdomen, in a straight line, branching off from the chest, under the ribs, to the hip, where it divided and extended to the right side of the spine, at the *small* of the back, and round the hip to the right groin, where the pain was much more acute than in any other part; if I except a small part close to the spine, which was very painful when touched, although the hand extended across the back at that spot, by giving support, enabled me to hold myself perfectly erect, which, without this, I was unable to do.' She also had some pain about the sigmoid flexure of the colon; but this was so slight that it was not much regarded.

"Her bowels during the long period of her ailments were very irregular. They were generally costive, but sometimes irritated, the evacuations being frequent, yet scanty, and occasionally attended with some degree of tenesmus. She had taken purgatives, but without relief; and had previously suffered from hæmorrhoids and slight sense of scalding in the urethra. The catamenia had not been materially deranged.

"The above are the most material particulars contained in a long history of her case, drawn up by herself at our request. On the first occasion of our seeing her, we examined carefully the abdomen, particularly over the cæcum and in the course of the colon; and we paid especial care to ascertain the existence of diseased liver. The region of the cæcum was unusually tumid, and very painful to the touch, with an inelastic hardness, extending in the course of the colon, under the right ribs, and across the epigastrium. Her countenance was dusky, and deficient in clearness. The tongue foul and the body emaciated. The cause of her ailments seemed to be perfectly evident; and we accordingly directed a pill, consisting of the blue-pill, with the aloes and myrrh pill, to be taken at bed-time every night; and a mixture of the compound infusions of senna and gentian, with the sulphate of magnesia and tincture of cardamoms, in the morning. These were continued daily, without intermission, excepting in as far as that calomel was combined with the aloes and myrrh every third or fourth night, for some time, until the morbid accumulations seemed to have been carried off. After a few days the evacuations became more copious and more morbid; and the quantity of disordered matters brought away astonished the patient. As these were removed, her health improved; the distressing symptoms gradually disappeared; and by continuing the pills and mixture, so as to procure at least two full evacuations daily, her recovery became complete."

A case is next related of a young lady, who had been remarkably healthy for the first ten years of her life, when she began to lose flesh without any visible causes. About the age of 12 years, when Mr. A. saw her, she was affected with severe spasms almost daily—her bowels were irregular, and, when she took purgatives, the motions were black and fetid. On examination, a very considerable fulness was observed in the region of the cæcum, and the whole abdomen was found to have a tumid and doughy feel, as if the bowels were loaded. The tongue was foul and spotted, being white, with little red spots. The appetite was good. The spasms came on suddenly, and sometimes when she appeared to be in the best spirits. They affected the chest and respiratory organs. A regular course of purgation was directed.

"Calomel, in six and eight grain doses, was given every night at bedtime, with an aperient draught in the morning, for six nights. The motions were extremely morbid, dark coloured, of clay consistence, and mixed with gritty matter. From these morbid motions having been increased in quantity, as the calomel and purgatives were repeated, the family were impressed with the conviction that the medicines taken were really increasing the disease; but as the spasmodic affection diminished in frequency and severity, as the accumulated matters were removed from the bowels, and as she was not at all weakened by the purgatives employed, but, on the contrary, strengthened, the purgative plan was continued for a month, occasionally intermitting the calomel, as this medicine was given entirely with a view of separating the viscid secretions which lined the alimentary canal, and facilitating their removal by the purgative exhibited in the morning. After this time, calomel combined with scammony, occasionally with aloes and the blue-pill, ipecacuanha, &c. were given, always avoiding the effects of calomel on the salivary glands; and the aperient was continued every morning. This plan was regularly persisted in for several months, varying, of course, the prescription according to circumstances; and the mass of *heavy, clay-like matter,*—of a dark-blue at times, sometimes quite black, at other times brown and green, with a sediment of a dark colour, having the appearance of sand,—which was brought away, can scarcely be conceived, except by those who saw it; and as this matter came away the child improved.

"This plan has now been continued for about two years; and although spasms occur once in six or seven months, the young lady is perfectly well. She grows, and is in good health and flesh. The abdomen is now perfectly elastic and natural, and the tumour at the cæcum entirely removed."

A case is next related of a young lady who had complained for several years of a sense of fulness and uneasiness in the right iliac region, with distention in the abdomen and oppression at the precordia. Head-ache, spasms, slight convulsions, hysteria, &c. were added to the list of anomalous symptoms. The bowels were costive, the catamenia regular. On examination of the region of the cæcum there was evident fulness, with tenderness there. Blue pill, aloes, myrrh, &c. were given at night, and the black draught in the morning. These remedies were continued regularly, a full dose of calomel being given every third or fourth night. The alvine evacuations were, at first, offensive and tenacious, but not abundant. They afterwards became more copious and morbid, of a dark brown and olive colour, and for several weeks continued so. The pain in the region of the cæcum becoming more acute, leeches were applied there, followed by poultices, with relief. Two month's perseverance in this plan was crowned with success, the anomalous symptoms all disappearing with the cause of them.

The subject of dysentery will require a separate article.

